# Intra-session and Inter-day Reliability of the Myon 320 Electromyography System During Sub-maximal Contractions

**DOI:** 10.3389/fphys.2018.00309

**Published:** 2018-03-29

**Authors:** Graeme G. Sorbie, Michael J. Williams, David W. Boyle, Alexander Gray, James Brouner, Neil Gibson, Julien S. Baker, Chris Easton, Ukadike C. Ugbolue

**Affiliations:** ^1^School of Science and Sport, Institute for Clinical Exercise and Health Science, University of the West of Scotland, Hamilton, United Kingdom; ^2^Division of Sport and Exercise Sciences, Abertay University, Dundee, United Kingdom; ^3^Oriam: Scotland's Sports Performance Centre, Heriot-Watt University, Edinburgh, United Kingdom; ^4^School of Life Sciences, Pharmacy, and Chemistry, Kingston University, Kingston upon Thames, United Kingdom; ^5^Department of Biomedical Engineering, University of Strathclyde, Glasgow, United Kingdom

**Keywords:** sEMG, ICC, squat, front raise, handgrip

## Abstract

Electromyography systems are widely used within the field of scientific and clinical practices. The reliability of these systems are paramount when conducting research. The reliability of Myon 320 Surface Electromyography System is yet to be determined. This study aims to determine the intra-session and inter-day reliability of the Myon 320 Surface Electromyography System. Muscle activity from fifteen participants was measured at the anterior deltoid muscle during a bilateral front raise exercise, the vastus lateralis muscle during a squat exercise and the extensor carpi radialis brevis (ECRB) muscle during an isometric handgrip task. Intra-session and inter-day reliability was calculated by intraclass correlation coefficient, standard error of measurement and coefficient of variation (CV). The normalized root mean squared (RMS) surface electromyographic signals produced good intra-session and inter-day testing intraclass correlation coefficient values (range: 0.63–0.97) together with low standard error of measurement (range: 1.49–2.32) and CV (range: 95% Confidence Interval = 0.36–12.71) measures for the dynamic-and-isometric contractions. The findings indicate that the Myon 320 Surface Electromyography System produces good to fair reliability when examining intra-session and inter-day reliability. Findings of the study provide evidence of the reliability of electromyography between trials which is essential during clinical testing.

## Introduction

Electromyography (EMG) is the study of electrical activity produced by skeletal muscles. EMG analysis has become an important tool in many areas of scientific and clinical research (Norali and Som, 2009). EMG signals can be recorded in many different ways; with electrodes being placed under the skin but over the muscle (subcutaneous EMG), in the muscles between the fibers (intramuscular EMG), or on the skin over the belly of the muscle (surface EMG) (Enoka, 2008). Surface EMG (sEMG) is a non-invasive technique that has been used to analyse muscle activity. The sEMG method has been used to diagnose muscle dysfunction for clinical purposes (Wakeling et al., 2007), provide insight into the neural control of gait (Byrne et al., 2007) and different muscular contraction types (Troiano et al., 2008). It can also be used to determine muscle activation levels when performing athletic actions. The usability of sEMG data however is dependent on the reproducibility of the signal detection both within and between recording sessions (Hashemi Oskouei et al., 2013).

Intra-session sEMG measurements largely show good relative reliability (intraclass correlation coefficient, ICC > 0.80) (Worrell et al., 1998; Dankaerts et al., 2004; Hashemi Oskouei et al., 2013; Jobson et al., 2013; Carius et al., 2015). During intra-session testing, variability of how the skin is prepared and electrode placement are excluded, therefore making the repeated measurements less variable (Carius et al., 2015). Intra-session reliability of the sEMG signal has been previously measured during isometric and dynamic contractions (Larsson et al., 1999; Pincivero et al., 2000; Larivière et al., 2004; Meskers et al., 2004; Hashemi Oskouei et al., 2013). Previous studies that have investigated sub-maximal isometric contractions during intra-session testing generally report good reproducibility of the sEMG signal (ICC > 0.80) (Allison et al., 1993; Larsson et al., 2003; Dankaerts et al., 2004). When investigating dynamic contractions, there are limited studies that compare the reproducibility of the sEMG signal during intra-session testing. The few studies that have investigated the sEMG signal during dynamic contractions report fair (ICC = 0.60–0.79) to good (ICC = 0.80–1.00) reproducibility for EMG amplitude and mean power frequency (Larsson et al., 1999; Dorel et al., 2008). Dorel et al. (2008) reported that no significant differences were found between test and retest for 10 lower limb muscles investigated during a cycling task. Larsson et al. (1999) also reported good levels of reproducibility (ICC > 0.80) during sub-maximal shoulder flexion movements when recording muscle activity from the deltoid muscle.

Studies examining inter-day reliability often report reduced ICC and increased coefficient of variation (CV) measures (Worrell et al., 1998; Hashemi Oskouei et al., 2013; Jobson et al., 2013). It has been suggested in the literature that skin preparation and electrode placement, even if care is taken to reposition electrodes, is a major influence on inter-day variance (Veiersted, 1991). Jobson et al. (2013) marked participants with henna markings in an attempt to replicate the electrode position for inter-day testing, however, this method still displayed variability within the sEMG signal (CV: 15.8–41.5%). Hashemi Oskouei et al. (2013) also reported poor inter-day reliability when testing various isometric handgrip forces (ICC < 0.60). With regards to inter-session reliability for dynamic movements, the literature is limited and contrasting (Hashemi Oskouei et al., 2013). Larivie et al. (2000) reported acceptable ICC values (range: 0.70–0.88) from the trunk muscles during lateral bending movements. However, Jobson et al. (2013) reported low reliability of the sEMG signal during cycling during inter-day testing (ICC < 0.60).

Literature discussing intra- and inter-session reliability often report ICC as a measure of relative reproducibility or CV as a measure of absolute reliability (Dankaerts et al., 2004; Hashemi Oskouei et al., 2013; Jobson et al., 2013). Standard error of measurement (SEM) is also often reported to quantify the absolute consistency of the measurement (Weir, 2016). Previous studies have conducted experiments using sEMG systems such as Delsys, Noraxon and Bortec (Dankaerts et al., 2004; Mathur et al., 2005; Hashemi Oskouei et al., 2013; Jobson et al., 2013; Carius et al., 2015). These systems are popular amongst researchers due to their proven reliability in peer reviewed research (Mathur et al., 2005; Auchincloss and McLean, 2009; Hashemi Oskouei et al., 2013; Jobson et al., 2013). This study was designed to enable future research to be conducted with the Myon 320 sEMG System. With the Myon AG Company being relatively new to the EMG market, a limited amount of research has been published using this system (Konrad and Tilp, 2014a,b; Rashid et al., 2015). Studies published previously have investigated stretching techniques in addition to engineering and textile related works. While these studies provide insightful information on the efficacy of the Myon 320 sEMG System, there is still a limited amount of biomechanical related research to support the reliability of the Myon 320 sEMG System as a useful tool kit for sEMG assessment. The reliability of the sEMG system that is employed during clinical and research trials is paramount in order to provide reliable and accurate findings in clinical settings, as it can be used to guide diagnosis or therapeutic option.

Therefore the aim of the study was to determine the intra-session and inter-day reliability of the Myon 320 sEMG System and Prophysics Software using dynamic and isometric sub-Maximum Voluntary Contraction (MVC).

## Methods

Fifteen healthy male participants (Mean ± SD: age 23 ± 3 years, stature 180.8 ± 7.5 cm, mass 80.6 ± 9.6 kg), who were physically active, with no history of knee, hip or shoulder surgery or neuromuscular conditions volunteered for this study. Participants were asked to refrain from physical activity 24 h prior to taking part in the experiment in order to avoid the effects of cumulative muscular fatigue. All participants completed a physical readiness questionnaire and consent form before participating in the study. Ethical approval was granted by the University of the West of Scotland, School of Science and Sport Ethics Committee.

Participants were required to attend the laboratory on two separate occasions. The length between each of the trials was required to be greater than 2 days but no longer than 10 days. At the first visit to the laboratory the participants were familiarized with the environment and the exercises prior to data collection. All visits were performed at the same time of day to minimize the effects of diurnal variation and any variation of the procedure. Experimental data preparation and collection was performed by the same researcher to eliminate researcher variation. The order in which the exercises were performed was randomized for all testing conditions.

**Figure 1:**
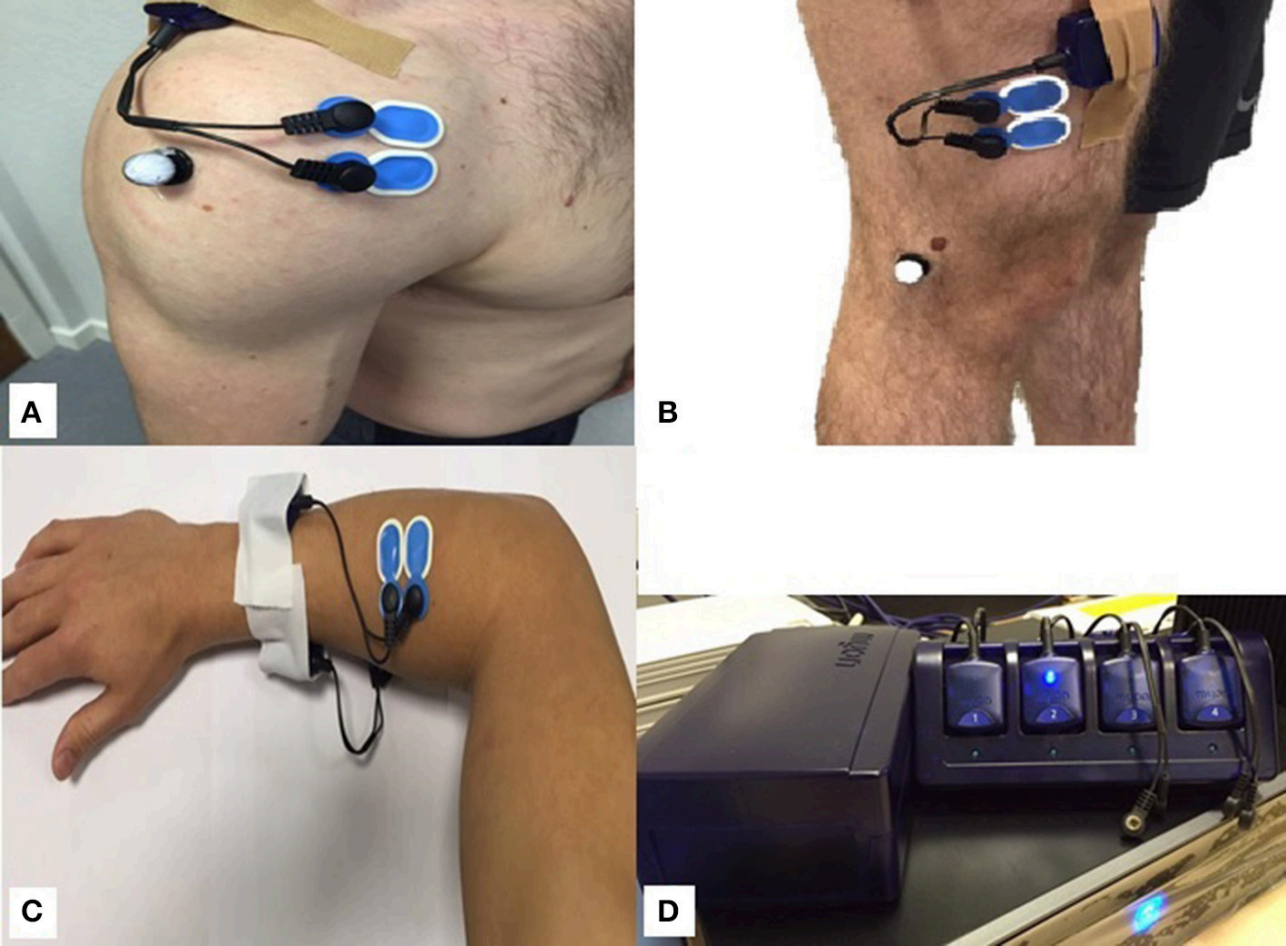
**(A)** Surface electrode connected to the anterior deltoid muscle; **(B)** Surface electrode connected to the vastus lateralis muscle; **(C)** Surface electrode connected to the extensor carpi radialis brevis muscle; and **(D)** Myon receiver box with transmitters sitting in cradle.

The sEMG activity was recorded using surface electrodes (AMBU, Cambridgeshire, UK) and a set of 6 Surface EMG Transmitters (Myon 320, Schwarzenberg, Switzerland). Prior to the sEMG data collection for the dynamic and isometric contractions, the skin was prepared by hair removal from the tested area, as well as skin abrasion and alcohol cleaning. This skin preparation procedure is essential in order to reduce the impedance of the interface between the skin and electrode. Pairs of sEMG electrodes were attached to the skin no more than 2 cm apart (center to center) over the dominant side of the anterior deltoid (AD) and vastus lateralis (VL) and extensor carpi radialis brevis (ECRB) muscles (Figure 1). To standardize the placement of the electrodes for the AD muscle, electrodes were placed one finger width distal and anterior to the acromion process, in the direction of the line between the acromion process and the thumb. For the VL muscle, electrodes were placed at two thirds on the line from the anterior superior iliac spine to the lateral side of the patella in the direction of the muscle fibers. These placement positions are in accordance with surface EMG for non-invasive assessment of muscles (SENIAM) guidelines. For the ECRB muscle electrode placement, a line was marked between the lateral epicondyle and the radial styloid process. The ECRB is located in the proximal half of the forearm, just lateral to the line (Basmajian, 1989; Sorbie et al., 2017). In order to ensure repeated sensor replacement between the days of testing, the location of the sensor was marked using a surgical skin demographic marking pen. Participants were instructed not to wash the markings off between the testing days.

For the dynamic contractions, two separate movement patterns were assessed: one for the upper and one for the lower extremity. For the upper extremity, a bilateral front raise, the lifting of an object in front of the body, exercise was performed with sEMG electrodes placed on the right AD muscle. All participants completed the bilateral front raise exercise with a calibrated 10 kg Taishan bumper plate weight (Taishan Sports Industry Group Co., Ltd, Leling, China). To execute the exercise, and standardize procedures, participants were instructed to stand with their feet shoulder width apart, holding the bumper plate with both hands around the waist line. From this position, participants raised the arms up in front of the body until the weight was directly above the head, with only a slight bend in the elbows, which was maintained throughout the movement. The shoulder at this stage of the exercise was required to be between 170 and 190° anterior to the body. The weight was then returned to the start position. Three trials of the front raise exercise were performed, with each trial consisting of three repetitions. Each of the three repetitions was performed at a rate of 4 s for the concentric phase and 4 s for the eccentric phase of the exercise, lasting a total of 24 s. This timing sequence was regulated through an interval timer, which enabled participants to move at a constant pace over the three trials, therefore making the movements more reliable. Between each trial, participants rested for 5 min to limit the effect of muscular fatigue. Retro-reflective markers were applied to the shoulder and hip area. This enabled the researchers to identify joint angles required to complete the movement.

For the lower extremity, sEMG data was collected from the right VL muscle during the unloaded squat exercise. During the squat, participants were instructed to have their feet shoulder width apart, whilst looking straight ahead. They were then asked to flex their knees between 100° and 80°, before returning to full knee extension, keeping their back as straight as possible. Three trials of the squat exercise were performed, with each trial consisting of three repetitions. The timing sequence as detailed above for the front raise exercise was implemented for the squat exercise, with the 5 min rest period between trials. Retro-reflective markers were applied to the hip, knee and ankle joints to enable the researchers to identify joint angles at the start and end of the exercise.

Isometric contractions were performed via three sub-MVC recordings from the right ECRB forearm muscle during a handgrip strength test. Following electrode placement and signals being verified, participants were seated with their right arm firmly strapped into the previously discussed experimental rig. Grip strength was recorded with a handheld dynamometer (Medical research Ltd digital analyzer, Leeds, UK). Firstly, participants were asked to perform two MVICs in order to normalize the sEMG data. Fifty percent of the greatest MVIC reading for the handheld dynamometer was selected for the three reproducibility trials. Participants had to build up to sub-MVCs in 3 s and then hold it for a further 3 s (Hoozemans and van Dieën, 2005). Participants were permitted to rest for 5 min between each trial to limit the effects of muscular fatigue on the ECRB muscle and surrounding forearm muscles.

The MVICs were recorded for 5 s for each muscle tested and was used as a reference for comparison of muscle activity during the bilateral front raise, squat and handgrip exercises (i.e., percentage of MVIC). Two 5 s MVICs were performed for each of the three muscles tested in the following positions; VL while the back was against the wall with 90° of knee flexion, AD while holding a 10 kg weight anterior to the body and shoulder flexed at 90°, and ECRB while seated with the right arm firmly strapped into a previously validated rig (unpublished data). In accordance with Hashemi Oskouei et al. (2013), the rig held the elbow at approximately 120° during repeated recordings, and kept the posterior side of the forearm stationary. The MVICs were performed prior to the front raise, squat and handgrip exercises on both testing days and controlled with the motion analysis device as described above.

All sEMG data was sampled at 1,000 Hz. During the processing procedures, all sEMG data was digitally filtered (20–400 Hz) in order to reduce transients and instrumentational noise and root mean squared (RMS) values calculated. For MVIC recordings, the maximum 1 s value across the 2 MVIC recordings for all muscles was identified and selected in order to normalize the bilateral front raise, squat and handgrip exercises. For the dynamic contractions, an RMS time window of 50 ms was employed. For the bilateral front raise exercises, the total duration of the movement was averaged and analyzed for reproducibility between the three trials. The identical procedure was also carried out for analysis of the squat exercise. In order for the researcher to analyse the dynamic exercises, kinematic data was recorded through the Vicon Bonita Motion System (Oxford Metrics Ltd, United Kingdom), sampling at a rate of 250 Hz. For the sub-MVIC handgrip test, an RMS time window of 100 ms was used and the 3 s 50% contraction was averaged to determine reproducibility of the three trials.

A two way random effects model with single and average ICC measures, with a 95% confidence interval, was used to measure the repeatability of the average normalized RMS sEMG signal during the intra-session testing. Inter-session reliability (ICC 2, 1) was determined by comparing the average normalized RMS sEMG muscle activity for the three trials for each exercise of both testing sessions. ICC, CV and SEM were obtained using the Statistical Package of Social Sciences (SPSS V 22.0). ICC was categorized as follows: good reliability: 0.80–1.00; fair reliability: 0.60–0.79; poor reliability: <0.60 (Sleivert and Wenger, 1994). Atkinson et al. (1999) also suggests a measurement tool is reliable if the ICC is above 0.800 and the CV is below 10%. SEM was used to express absolute reliability of the measure. The CV and the SEM were calculated as follows:

$$\begin{array}{l} CV=\frac{\text{SD}}{\text{Mean}}\;\times 100\%\;SEM\left(x\right)=SD\;\sqrt{1-r} \end{array}$$

Calculation acronyms: Coefficient of variation (CV), Standard deviation (SD), Reliability (r), Standard error of the measurement (SEM).

## Results

All participants successfully completed the required movements during the dynamic bilateral front raise and squat exercises. During the isometric handgrip task all participants, achieved 50% (±5%) of their MVIC value.

The average normalized RMS sEMG data between participants from the AD muscle over the three sub-MVC trials of the bilateral front raise exercise displayed good within-day reliability [ICC (2, 1) = 0.97] and an acceptable CV of 4.73% (95% CI = 1.35–9.79). The average muscle activation between participants was 66.05% ± 20.15 for the sub-MVC bilateral front raise exercise. SEM between participants was 2.06. Inter-day reliability for the average normalized RMS sEMG for the AD during the bilateral front raise exercise produced good reliability [ICC (2, 5) = 0.94] and an acceptable CV of 3.86% (95% CI = 0.82–7.46). The average muscle activation for inter-day testing between participants was 65.85% ± 18.51 for the sub-MVC front raise exercise. The SEM between participants during inter-day testing was 1.49.

For the squat exercise, the average normalized RMS sEMG data from the VL muscle over the three sub-MVC trials displayed good within-day reliability [ICC (2, 1) = 0.95] and an acceptable CV of 5.73% (95% CI = 1.48–8.94). The average muscle activation during intra-day testing between participants was 67.87% ± 21.25 for the sub-MVC squat exercise. SEM between participants was 2.32. Inter-day reliability for the average normalized RMS sEMG from the squat exercise produced good reliability [ICC (2, 5) = 0.93] and an acceptable CV of 4.77% (95% CI = 1.62–7.52). The average muscle activation for inter-day testing between participants was 67.10% ± 20.63 for the sub-MVC squat exercise. The SEM between participants during inter-day testing was 1.84.

For the isometric handgrip test the average normalized RMS sEMG data from the ECRB forearm muscle over the three trials displayed good within-day reliability [ICC (2, 1) = 0.87] and an acceptable CV of 5.89% (95% CI = 0.36–12.36). The average muscle activation between participants was 45.98% ± 8.82 for the handgrip test. SEM between participants was 1.57. On the other hand, inter-day relative reliability was fair during single isometric contractions [ICC (2, 5) = 0.63]. CV also increased to 7.18% (95% CI = 3.40–12.71). The average muscle activation for inter-day testing between participants was 45.91% ± 8.09 for the sub-MVIC handgrip test. The SEM between participants during inter-day testing for the isometric contraction was 1.93.

## Discussion

This is the first study to assess the reliability of the Myon 320 sEMG system during low velocity controlled movements, such as those routinely used in rehabilitation. The researchers investigated intra-session and inter-day reliability during sub-maximal dynamic and isometric contractions while recording sEMG measurements using the Myon 320 sEMG System. The main findings were that the Myon 320 sEMG System displayed good reliability associated with normalized RMS sEMG measures (ICC > 0.80) for intra-session and inter-day testing during dynamic sub-MVC. During 50% MVIC contractions the Myon 320 sEMG System produced good intra-session repeated measures (ICC > 0.80) and fair inter-day measures (ICC 0.60–0.79). The normalized RMS sEMG within the group of participants in the study displayed a strong correlation with the 50% MVIC during the intra (45.98%) and inter-day (45.91%) testing.

The high intra-session ICC for the normalized RMS sEMG signal during the bilateral front raise and squat exercises presented in the current study is consistent with previously published literature (Worrell et al., 1998; Larsson et al., 1999; Jobson et al., 2013). Larsson et al. (1999) reported that reproducibility of the RMS sEMG signal was good and clinically acceptable during dynamic forward flexion exercises when recording muscle activity from the deltoid muscle. Similar to the current study, Worrell et al. (1998) used normalized RMS sEMG and reported good reliability when recording sEMG from the VL muscle during an unweighted lateral step exercise (LSU) (ICC = 0.91). During the LSU the VL muscle had an activation percentage of 63% ± 24 MVIC. These reported reliability and muscle activation results are similar to the current studies results (ICC = 0.95) (68% ± 21 MVIC). However, even with these good ICC reliability measures during dynamic contractions, two participants displayed high variability between the three trials performed on each of the testing days. The researchers suggest these inconsistences are a result of increased perspiration levels from the participants. This increased perspiration caused the AMBU surface electrodes to move or detach leading to artifacts within the sEMG signal. The movement of the surface electrodes was more noticeable during the dynamic contractions than the isometric contractions. These views are supported by Rashid and colleagues who also documented problems with perspiration when testing with the Myon 320 sEMG System (Rashid et al., 2015). In addition, signal artifacts were also displayed within one participant's data set when testing the VL during the squat exercise when the cable connection (length: 13 cm) between the transmitter box and surface electrode came in contact with the participants shorts. This problem was solved by taping the shorts above the VL muscle. The taping in no way restricted the participants' movements during the squat exercise.

When comparing intra-session to inter-day testing for dynamic exercises, the present study reported reduced ICC measures, however, these were still within the suggested range for good reliability (ICC > 0.80). The literature for inter-session reliability is somewhat contrasting to the findings of the current study. Worrell et al. (1998) reported poor ICCs during a dynamic lateral step task. Jobson et al. (2013) results also displayed poor ICC measures during cycling. One explanation for the contrasting results could be the highly standardized range of motion (ROM) of each of the dynamic exercises performed in this study. This could have resulted in more consistent measures. It could also be suggested that the step (Worrell et al., 1998) and cycling (Jobson et al., 2013) reliability tests were performed at a higher velocity than the squat and bilateral front raise tests performed in this study, which could have resulted in the contrasting findings. In addition to this, differences in findings could be attributed to surface electrode placement repeatability on the specified muscles and not the exercises performed within the different protocols.

With regards to isometric contractions, the good ICC (0.87) values for the normalized sEMG RMS data during intra-session testing in the current study is consistent with previously published research (Dankaerts et al., 2004; Hashemi Oskouei et al., 2013). Hashemi Oskouei et al. (2013) reported good intra-session ICC of 0.90 when recording muscle activity from the forearm flexor muscles during gripping tasks. Good within-day reliability (ICC = 0.91) has also been reported during MVIC trunk exercises (Dankaerts et al., 2004).

With regards to inter-session reliability during isometric contractions in this study, it would appear that reapplying the electrodes on a subsequent day reduces the repeatability of the normalized RMS sEMG signal. These findings are in agreement with previous published literature (Hashemi Oskouei et al., 2013) in which the removal and replacement of the surface electrodes to the flexor muscles of the forearm resulted in fair to poor inter-day reliability of the sEMG signal. A possible explanation for the reduction in ICC results during the isometric contractions within the two studies could be caused by the size and proximity of the flexor and extensor muscles of the forearm (Hägg and Milerad, 1997). The forearm area is comprised of many adjacent small muscles, therefore increasing the possibility of EMG cross-talk. When measuring muscle activity for the ECRB muscle during the current study an inter-electrode distance of 2 cm was selected which is in accordance with previous literature (Hägg and Milerad, 1997; Sorbie et al., 2017), however, a reduced inter-electrode distance should be considered in future reliability research in order to reduce potential cross-talk. The potential for surface electrodes to record signals from multiple extensor forearm muscles is a concern (Gallina and Botter, 2013). These suggestions are supported by Dankaerts et al. (2004) who reported good ICC values for inter-day reliability when testing muscles with a larger belly circumference (trunk muscles) than that of the forearm muscles. It could also be suggested that these contrasting findings could be the result of difficulty in controlling fatigue in the smaller forearm muscles. As a result of these concerns, isometric contractions from larger muscle groups are preferred when using the Myon 320 sEMG System. In addition to this, the current study is limited with regards to measuring dynamic contractions from the forearm muscles. As a result of this limitation, the reliability of dynamic contractions from forearm muscles when using the Myon 320 sEMG System should be considered in future.

## Conclusion

When using the Myon 320 sEMG System, the present study shows that it is possible to obtain good reliability for normalized RMS sEMG during intra-session and inter-day testing during dynamic sub-MVC, when exercises are performed at low velocities. This study also highlights the fair reproducibility of the normalized RMS sEMG from the extensor muscles of the forearm during a handgrip task during inter-session testing, which is in agreement with previously published literature. Therefore, the current study demonstrates that the Myon 320 sEMG System is a reliable sEMG measurement tool, for low velocity controlled movements.

## Author contributions

All authors contributed to the development of this manuscript. UU, GS, and CE were involved in the experimental design. GS, MW, DB, and AG were involved in the data collection. UU, GS, MW, DB, and AG were involved with the data processing and analyses. UU, GS, CE, MW, JB, JSB, NG were involved in the writing and proof reading of the manuscript.

### Conflict of interest statement

The authors declare that the research was conducted in the absence of any commercial or financial relationships that could be construed as a potential conflict of interest.
